# Supplementary material for: Pulmonary lesions following inoculation with the SARS-CoV-2 Omicron BA.1 (B.1.1.529) variant in Syrian golden hamsters
Source: Emerg Microbes Infect. 2022 Jul 17;11(1):1778–86. doi: 10.1080/22221751.2022.2095932 (PMC9295819; doi:10.1080/22221751.2022.2095932)
Supplement: Supplemental Material [file TEMI_A_2095932_SM9406.zip › Supplement_MRissmann_revised.docx]

**Supplemental materials**

**Animals and Ethical Statement**

Research involving animals was conducted in compliance with the Dutch legislation for the protection of animals used for scientific purposes (2014, implementing EU Directive 2010/63) and other relevant regulations. The licensed establishment where this research was conducted (Erasmus MC) has an approved OLAW Assurance # A5051-01. Research was conducted under a project license from the Dutch competent authority and the study protocol (#17-4312) was approved by the institutional Animal Welfare Body. Animals were housed in groups of 2 animals in filter top cages (T3, Techniplast), in Class III isolators allowing social interactions, under controlled conditions of humidity, temperature and light (12-hour light/12-hour dark cycles). Food and water were available ad libitum. Animals were cared for and monitored (pre- and post-infection) daily by qualified personnel. All animals were allowed to acclimatize to husbandry for at least 7 days. For unbiased experiments, all animals were randomly assigned to experimental groups. Experimental groups consisted of n=4 animals. Statistical power was calculated with the Statmate software for determining sample sizes to compare 2 means (unpaired t-test)' and based on a significance level α of 0.05, a power β of 80% and the assumption that a strain-dependent effect on pathogenesis or shedding will be observed in 50% of the animals. A standard deviation of 25% was expected.

The animals were anesthetized (3-5% isoflurane) for all invasive procedures. Hamsters were euthanized by cardiac puncture under isoflurane anesthesia and cervical dislocation.
