# Supplementary figures and images for: Pulmonary lesions following inoculation with the SARS-CoV-2 Omicron BA.1 (B.1.1.529) variant in Syrian golden hamsters
Source: Emerg Microbes Infect. 2022 Jul 17;11(1):1778–86. doi: 10.1080/22221751.2022.2095932 (PMC9295819; doi:10.1080/22221751.2022.2095932)

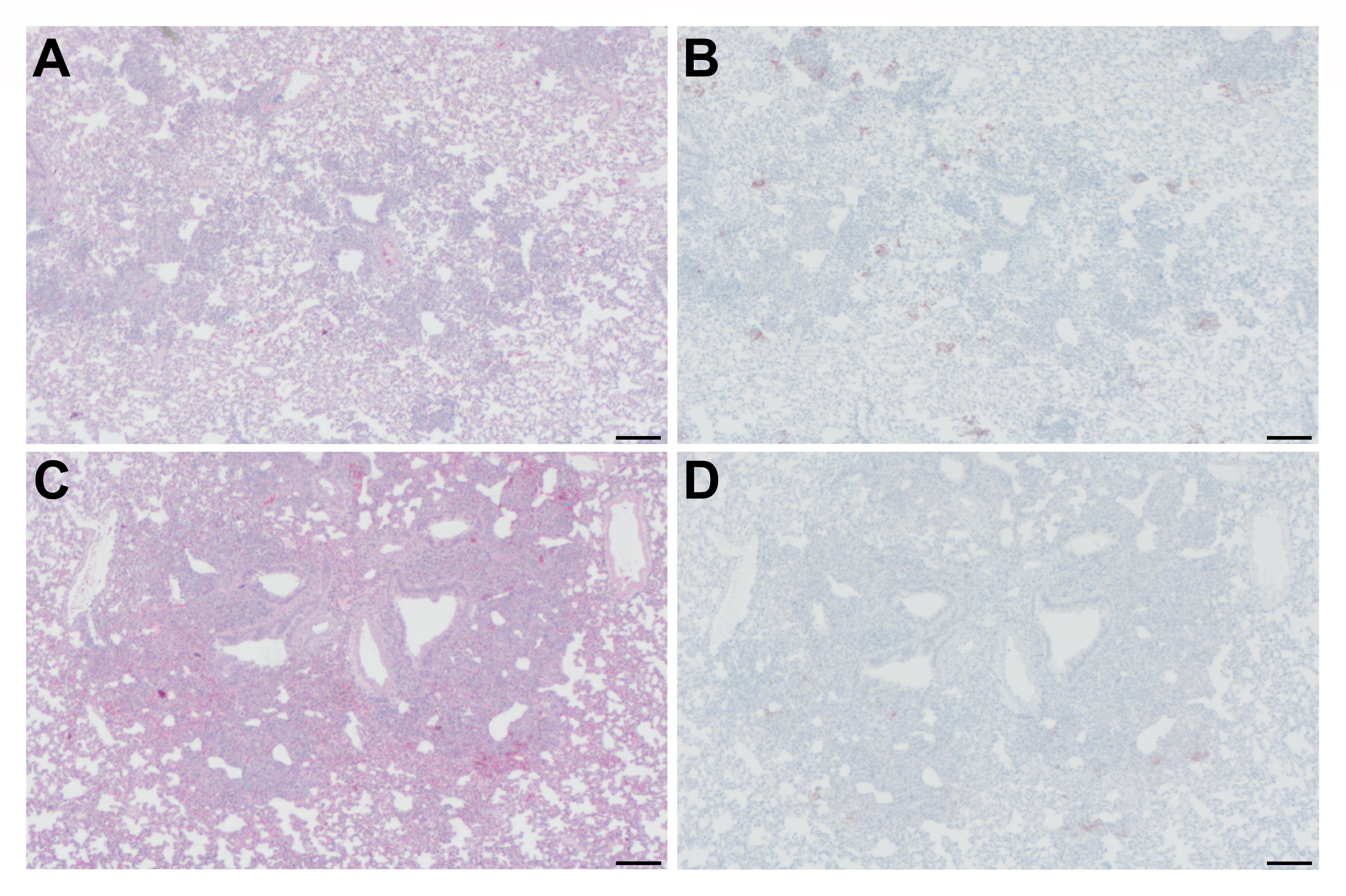

Supplement: Supplemental Material [file TEMI_A_2095932_SM9406.zip › SFig1.jpg]
